# Health-related quality of life in older women with injuries: a nationwide study

**DOI:** 10.3389/fpubh.2023.1149534

**Published:** 2023-05-25

**Authors:** Yeunhee Kwak, Jung-Won Ahn

**Affiliations:** ^1^Red Cross College of Nursing, Chung-Ang University, Seoul, Republic of Korea; ^2^Department of Nursing, Gangneung-Wonju National University, Wonju-si, Gangwon-do, Republic of Korea

**Keywords:** injury, older women, health-related quality of life, subjective health status, stress

## Abstract

**Objectives:**

This study aims to describe the health-related quality of life (HRQoL) and influencing factors of older women who experienced injuries.

**Methods:**

This study is a secondary analysis of data from 4,217 women aged 65 years or older sampled from the Korea National Health and Nutrition Examination Survey (KNHANES) (2016–2020) database. Two-way analysis of variance was used to analyze the data.

**Results:**

The mean HRQoL scores of older women with and without injuries were 0.81 ± 0.19 (*n* = 328) and 0.85 ± 0.17 (*n* = 3,889), respectively, which were significantly different (*p* < 0.001). The results of multiple regression analysis revealed that working, physical activity, BMI, osteoarthritis, stress, and subjective health status significantly affected the HRQoL of older women with injuries, and the explanatory power of the model was 29%.

**Conclusion:**

The results of this study on factors affecting HRQoL can contribute to the understanding of the experience of older women with injuries and can be used as a reference to develop health promotion programs.

## Introduction

Accidents commonly occur among older adults and can cause significant health issues in aging populations (1). In 2019, the annual number of injuries in Korea was 3.71 million and had been increasing yearly. As age of the population increases, the number of hospitalized patients with injuries tends to increase, with 50% of those aged 65 or older hospitalized due to injuries (2). Injuries, hospitalizations, and mortality rates are higher in men than in women. However, among adults over 75 years of age, women were 1.3 times more likely to experience injuries, hospitalizations, and mortality rates than men. Injuries are a major cause of hospitalization and a significant public health threat for women, and they inflict a heavy socioeconomic burden on older adults (2, 3). Injury-related trauma can worsen older adults’ physical, mental, and social well-being, significantly increasing hospitalization, morbidity, mortality, and healthcare costs (4, 5). Previously, accidents were seen as unpredictable occurrences; however, there has been a recent shift in awareness toward accidents being preventable through nationwide interventions and individual lifestyle changes (6).

An injury refers to “harmful consequences in terms of physical and mental health that occur as a result of intentional or unintentional accidents” (7). Injuries also affect an individual’s emotional state and can reduce their overall quality of life through loss of self-confidence and increased social isolation (1, 8). As the older adult population increases, the threats to their health and quality of life increase along with the associated socioeconomic costs, creating a major socioeconomic and socio-emotional problem (9, 10).

Health-related quality of life (HRQoL), defined as subjective satisfaction with life in physical, psychological, mental, and socioeconomic domains, is becoming increasingly important to older adults with their increase in life expectancy (11, 12). Sex, socioeconomic level, physical and mental health, the ability to perform daily activities, subjective health status, and health behaviors were found to be the factors that most significantly influenced the HRQoL of older adults (11, 13, 14). Notably, the HRQoL of older women was lower than that of men (1, 15). After the death of a spouse, older adult women have a more challenging time than men. This is due to poor health, poverty, and depression (16).

Factors associated with HRQoL in older women vary (15). Living alone decreases the HRQoL for older adults that have a low economic status and education level (10, 14). Health behaviors such as consuming alcohol, cigarette smoking, and exercising also affect the HRQoL (1, 16). The HRQoL was lower in patients with depression or chronic conditions, such as hypertension or diabetes (15, 17). It was also lower in patients with reduced muscle mass or limited physical activity (14, 18).

This study investigated the HRQoL of older women who experienced injuries using a dataset from the Korea National Health and Nutrition Examination Survey (KNHANES) from 2016 to 2020 in order to gain a better understanding of their quality of life. The purpose of this study was to evaluate the HRQoL of older women with and without injuries, to determine whether there is a difference in the HRQoL among older women (measured by the EQ-5D index) with and without injuries based on demographic characteristics, health behavior, and health status, and to identify factors associated with the HRQoL of older women with injuries.

## Materials and method

### Study design

The KNHANES has been performed to identify the state of Korean health and nutrition since 1998 by the Korea Disease Control and Prevention Agency (KDCA). This cross-sectional study is a secondary analysis of data from the KNHANES from 2016 to 2020. The KNHANES is a nationally representative database for cross-sectional studies targeting non-institutionalized Koreans and consists of health, nutrition, and physical examination surveys. The health and nutrition surveys were conducted as one-on-one interviews and self-report methods. A professional survey team administered the physical examination survey from the KDCA.

### Study setting and participants

A representative sample of the entire Korean population was obtained using a stratified multistage cluster sampling and probability design (19, 20). According to the KNHANES data from 2016 to 2020, of the 4,217 women aged 65 years or older, 328 (7.8%) of them had experienced injuries. Missing data from the questionnaire and test values were excluded from the final analysis. The KNHANES was approved by the Institutional Review Board of the KDCA.

### Variables

#### Demographic characteristics

The demographic variables of the subjects included age, education level, whether they lived alone, income level, occupational status, and area of residence. Education level was classified as elementary or lower, middle, high school, and college or higher. Income was divided into quartiles based on the equivalent income, which was defined as the household income adjusted for the number of household members using the formula which was defined as the household income divided by the square root of the number of household members. The residence was divided into urban and rural areas.

#### Injury occurrence

Injury occurrence was measured by recording the responses to the question, “Have you had an accident or poisoning incidence that required treatment at an acute or primary care facility such as an emergency room, public health center, or health clinic within the past year?” The responses were classified as yes or no, and injuries that only required self-care or over-the-counter medicine were excluded. There were nine categories for the mechanisms of injury, including transportation accidents, falls or slip, bumping against objects, lacerations/stings/cuts/piercing wounds, burns, suffocation, drowning, and poisoning.

#### HRQoL

The HRQoL was measured using the EQ-5D index (EuroQol-5 Dimension) developed by the EuroQol Group (21). The EQ-5D is an instrument used for measuring HRQoL. It was developed as a simplified measurement of overall health to be used for clinical and economic evaluations (22). The EQ-5D consists of five dimensions: mobility, self-care, usual activities, pain/discomfort, and anxiety/depression. In this study, the weighting of the KDCA, which expresses health status as a value from −0.171 to 1 in consideration of the characteristics of Koreans, was applied. The weights were as follows: EQ_5D = 1 − (0.05 + 0.096 × M2 + 0.418 × M3 + 0.046 × SC2 + 0.136 × SC3 + 0.051 × UA2 + 0.208 × UA3 + 0.037 × PD2 + 0.151 × PD3 + 0.043 × AD2 + 0.158A × D3 + 0.05 × N3) (19). The possible score of the EQ-5D index ranges from −1 to 1.

#### Health-related behavior

Smoking was classified as “Yes” if the subject smoked or “No” if they did not. Drinking was defined as “Yes” if the subject drank more than once a month or “No” if they drank less than once a month. Physical activity was classified as “Yes” if the subject performed moderately intense physical activity for longer than 2 h and 30 min per week, highly intense physical activity for longer than 1 h and 15 min per week, or moderate to intense physical activity. The others were classified as “No” (23, 24). Sleep was classified as less than 7 h or more than 7 h based on the answer to the question, “How many hours do you usually sleep a day?”

#### Health status

Health status included hypertension, diabetes, osteoporosis, osteoarthritis, body mass index (BMI), stress, and subjective health status. Physical health characteristics, including hypertension, diabetes, osteoporosis, and osteoarthritis, were based on the physician’s diagnosis. BMI was calculated using the formula: weight (kg)/height (m)^2^. The results were divided into BMI over 25 (overweight), BMI between 18.5 and 24.9 (healthy weight), and BMI less than 18.5 (underweight). Stress was categorized as “Yes” or “No” based on the answers to the question, “How much stress do you feel in your daily life?” Stress was categorized as “Yes” for “extremely stressed” or “very stressed” and “No” for “a little stressed” or “barely stressed.” Subjective health status was assessed using self-reports as a reliable indicator of an individual’s overall health level. Responses of “very good” and “good” to the question “How do you usually feel about your health?” were categorized as having a good subjective health status, while “average” and “bad” or “very bad” were classified as having an average or bad subjective health status, respectively.

#### Statistical analysis

Statistical analysis was performed using SAS software 9.3 (SAS Institute Inc., Cary, NC, United States) to run a complex sample design based on the results of data analysis of the survey data; this provided the sampling weights of the KNHANES and nationally representative estimates. All data were presented as mean ± SD for continuous variables. The difference in the HRQoL for different groups of demographic characteristics, health-related behavior, and health status was examined by two-way analysis of variance to determine how two independent variables (general characteristics and injury occurrence) affect the HRQoL. Finally, multiple regression analysis was performed after adjusting for the general characteristics of the patients who showed significant differences to determine the relationship of the HRQoL with health-related behavior and health status. Statistical significance was determined by a *p* value less than 0.05.

## Results

The HRQoL score of older women with injuries was 0.81 ± 0.19, and the score of older women without injuries was 0.85 ± 0.17; there was a significant difference between the two groups (*t* = 65.11, *p* < 0.001). Two-way analysis of variance showed that the level of HRQoL was significantly different between women with and without injuries depending on their demographic characteristics, health-related behavior, and health status. There was a significant difference in the HRQoL score of older women with and without injuries according to age, education level, living status (alone or not alone), level of income, working status, and residence area (*p* < 0.001). In terms of health-related behavior, there was a significant difference in the HRQoL score according to the participants’ cigarette smoking habit, alcohol drinking habit, physical activity, and sleep hours (*p* < 0.001). However, there was no significant difference in the HRQoL score according to alcohol drinking habit between women with and without injuries. In terms of health status, the HRQoL score was different according to the presence or absence of hypertension, diabetic mellitus, osteoporosis, osteoarthritis, and stress (*p* < 0.001). In addition, there was a difference in the HRQoL between different BMI groups, which showed that the normal BMI group had a higher HRQoL score compared with the scores of the lower and higher BMI groups (*p* < 0.001). Finally, there was a significant difference in the HRQoL score according to the subjective health status, and the group with bad subjective health status had a low HRQoL score (*p* < 0.001) (Table 1).

**Table 1:** HRQoL according to the characteristics of older women with injuries (*n* = 4,217).

Variables	Categories		EQ-5D index		*t* or *F* (*p*-value)
			Older women with injuries (*n* = 328) *M* (SD)	Older women without injuries (*n* = 3,889) *M* (SD)	
Total			0.81 (0.19)	0.85 (0.17)	65.11 (<0.001)
Demographic	Age (year)	65–74	0.82 (0.20)	0.88 (0.15)	112.13 (<0.001)^†^
		≥75	0.77 (0.21)	0.83 (0.18)	
			40.09 (<0.001)^‡^		
	Education level	Elementary school or less	0.77 (0.21)	0.85 (0.17)	49.32 (<0.001) ^†^
		Middle school	0.84 (0.18)	0.89 (0.14)	
		High school	0.88 (0.15)	0.92 (0.12)	
		College or higher	0.86 (0.16)	0.93 (0.10)	
			42.07 (<0.001)^‡^		
	Living alone	Yes	0.77 (0.24)	0.84 (0.18)	41.51 (<0.001)^†^
		No	0.82 (0.18)	0.87 (0.15)	
			39.43 (<0.001)^‡^		
	Income	Lower	0.78 (0.22)	0.84 (0.18)	34.84 (<0.001)^†^
		Lower-middle	0.83 (0.18)	0.88 (0.14)	
		Upper-middle	0.84 (0.19)	0.90 (0.14)	
		Upper	0.85 (0.18)	0.91 (0.13)	
			41.08 (<0.001)^‡^		
	Working	Yes	0.88 (0.20)	0.88 (0.16)	22.83 (<0.001)^†^
		No	0.77 (0.19)	0.85 (0.17)	
			39.46 (<0.001)^‡^		
	Residence area	Urban	0.82 (0.19)	0.87 (0.16)	36.59 (<0.001)^†^
		Rural	0.78 (0.20)	0.84 (0.18)	
			39.39 (<0.001)^‡^		
Health-related behavior	Cigarette smoking	Yes	0.74 (0.18)	0.82 (0.16)	6.61 (0.010)^†^
		No	0.81 (0.20)	0.87 (0.18)	
			39.11 (<0.001)^‡^		
	Alcohol drinking	Yes	0.80 (0.21)	0.87 (0.17)	0.07 (0.080)^†^
		No	0.81 (0.20)	0.87 (0.16)	
			39.05 (<0.001)^‡^		
	Physical activity	Yes	0.87 (0.16)	0.90 (0.13)	101.96 (<0.001)^†^
		No	0.78 (0.21)	0.85 (0.17)	
			37.99 (<0.001)^‡^		
	Sleep hours	<7	0.80 (0.20)	0.86 (0.17)	11.63 (<0.001)^†^
		≥7	0.82 (0.21)	0.88 (0.15)	
			39.16 (<0.001)^‡^		
Health status	Hypertension	Yes	0.79 (0.21)	0.85 (0.17)	49.96 (<0.001)^†^
		No	0.82 (0.20)	0.89 (0.15)	
			39.51 (<0.001)^‡^		
	Diabetic Mellitus	Yes	0.80 (0.21)	0.84 (0.17)	28.77 (<0.001)^†^
		No	0.81 (0.20)	0.87 (0.16)	
			39.31 (<0.001)^‡^		
	Osteoporosis	Yes	0.79 (0.20)	0.84 (0.17)	63.53 (<0.001)^†^
		No	0.82 (0.21)	0.88 (0.15)	
			39.64 (<0.001)^‡^		
	Osteoarthritis	Yes	0.76 (0.21)	0.83 (0.17)	168.18 (<0.001)^†^
		No	0.84 (0.19)	0.89 (0.15)	
			40.59 (<0.001)^‡^		
	BMI (kg/m^2^)	<18.5	0.65 (0.26)	0.79 (0.23)	33.36 (<0.001)^†^
		18.5–24.9	0.82 (0.20)	0.88 (0.15)	
		25<	0.80 (0.20)	0.85 (0.16)	
			39.66 (<0.001)^‡^		
	Stress	Yes	0.72 (0.23)	0.79 (0.20)	251.79 (<0.001)^†^
		No	0.83 (0.19)	0.89 (0.14)	
			39.90 (<0.001)^‡^		
	Subjective health status	Good	0.93 (0.10)	0.94 (0.09)	597.95 (<0.001)^†^
		Average	0.87 (0.15)	0.91 (0.11)	
		Bad	0.70 (0.22)	0.76 (0.19)	
			50.15 (<0.001)^‡^		

† Adjusted for injury and no injury experience to analyze the difference in the EQ-5D index score according to general characteristics.

‡ Adjusted for general characteristics to analyze the difference in the EQ-5D index score between women with and without injuries.

SD, standard deviation.

Table 2 shows the results of analyzing the factors related to the HRQoL of older women with injuries. For multiple regression analysis, age, education level, income, and residence area were analyzed following statistical adjustment. Physical activity (*ß* = 0.05, *p* = 0.035) and normal BMI (*ß* = 0.15, *p* = 0.006) had a positive effect on HRQoL; however, not currently working (*ß* = −0.06, *p* = 0.018), osteoarthritis (*ß* = −0.04, *p* = 0.040), stress (*ß* = −0.06, *p* = 0.019), and bad subjective health status (*ß* = −0.19, *p* < 0.001) had a negative effect on the HRQoL of older women with injuries (Table 2).

**Table 2:** Factors affecting the HRQoL of older women with injuries^†^ (*n* = 328).

Variables	*ß*	SE	*t*	*p*
Intercept	0.87	0.09	9.29	<0.0001
Working (no)	−0.06	0.23	−2.38	0.018
Cigarette smoking (yes)	−0.02	0.07	−0.23	0.819
Alcohol drinking (yes)	−0.01	0.02	−0.32	0.747
Physical activity (yes)	0.05	0.02	2.12	0.035
Hypertension (yes)	−0.01	0.02	−0.09	0.925
Diabetic Mellitus (yes)	0.01	0.02	0.26	0.792
BMI (18.5–24.9 kg/m^2^)	0.15	0.05	2.75	0.006
Osteoporosis (yes)	0.02	0.02	0.79	0.429
Osteoarthritis (yes)	−0.04	0.02	−2.06	0.040
Stress (yes)	−0.06	0.02	−2.34	0.019
Subjective health status (bad)	−0.19	0.03	−5.98	<0.001
*F* (*p*) = 6.65 (<0.001) *R*^2^ = 0.34, Adjusted *R*^2^ = 0.29				

† Adjusted for age, education, income, and residence area.

SE, standard error.

## Discussion

This study analyzed the HRQoL of older women who experienced injuries using raw data from the KNHANES over 5 years (2016–2020). To evaluate the difference in the HRQoL score between older women with and without injuries, general characteristics were adjusted by two-way analysis of variance, and the HRQoL score in the injured group was significantly lower. Previous studies have reported a lower HRQoL for those who experienced a fall (8, 25). Falls are negatively associated with HRQoL, independent of chronic diseases or conditions (8). The quality of life of older women tends to decrease after experiencing an injury, making them a high-risk group that needs care support in relation to their social and healthcare services. Based on the results of this study, the HRQoL of women aged 75 years or older was significantly lower than that of women aged 65–74 years, and it was the lowest in the injured group. Previous studies have also indicated that falls become more frequent and the HRQoL decreases with an increase in age (1, 8, 10, 14). The findings suggest that older women with injuries need more attention and government support.

The results of this study are consistent with previous studies showing that the HRQoL was high in people with a high education level (10, 26, 27). People with higher education levels are reported to have a higher perceived quality of life relating to their socioeconomic affluence and healthy activities. Based on this information, we propose creating various lifelong education programs provided by welfare centers in which older adults can participate in continuing education. In addition to education level, older women who were currently working tended to have a higher level of HRQoL. Having a job was a positive factor for HRQoL. The education level of older adults affects their quality of life, mediated by economic activity, employment status, health status, and social relationships; however, it can also directly affect the quality of life regardless of sex or age (26, 28).

Participation in economic activities may be the most influential factor in improving the quality of life of older adults. It provides a space for social participation, fulfills basic living needs, assists people in recovering from their sense of loss of a role relating to a job, and increases physical and mental satisfaction (29). Job-related activity assists older adults in recovering their health and extending their life expectancies beyond various chronic diseases they suffer from Kwak and Kim (28). Older adults often work is to build social networks, not to support their families. Thus, establishing an employment support system for older job seekers is necessary to support their social networks and improve their quality of life.

Living alone was a factor that lowers the quality of life of older adult women with injuries in this study. According to studies investigating older men and women, marital status affected just the health of men (14, 30, 31). However, this study also showed that the HRQoL score of injured old women was lower in the absence of a spouse or cohabitant, indicating that injury experience may contribute to HRQoL decline. Many older adults living alone experience difficulties, such as health problems and poverty, and their ranks are expected to increase (32). In Korea, home-visiting healthcare services for older adults living alone are operated only by public health centers in each local government and are mostly led by nurses (33). To address a growing demand for services, it will be necessary to expand these services nationwide with a multidisciplinary team of service providers, including doctors, physical therapists, and pharmacists. Utilizing welfare facilities, health support centers, and senior citizen centers in various ways can provide places for older adults living alone or after an injury to form a family living community and promote interaction among older adults. It will encourage relations between older adults with various health risks and improve their quality of life.

In this study, there was a difference in the HRQoL depending on the region of residence. It has been reported that older adults living in cities had a higher quality of life than older women living in rural areas (10, 27), indicating that a high HRQoL might be more common in socioeconomically developed areas. This could be caused by the unequal distribution of healthcare services and health-related welfare services between urban and rural areas. Population aging is occurring at a rapid rate in Korea. Notably, the aging rate in rural areas is much steeper due to younger people leaving to live in the cities (34). Thus, government policies should consider this when developing healthcare services for rural areas.

Among older women with injuries, the HRQoL was low for those who could not participate in physical activities. Multiple regression analysis revealed that physical activity was a positive influencing factor of HRQoL, and osteoarthritis, a chronic disease with a negative effect on physical activity, was found to be a significant negative influencing factor for injured old women. According to the current injury occurrence in Korea, the number of patients is higher for older adults. Among patients over 75 years of age, there were 1.3 times more female patients than male patients. Interrupted physical activity after an injury reduces the HRQoL. Chronic diseases, weakness, and reduced daily activities, which older adults commonly experience, limit mobility, increase their dependence on others, and negatively impact their quality of life (11). Additionally, reduced muscle mass and physical activity limitations are associated with a low HRQoL (18). Regular exercise, particularly physical activity for more than 5 days a week, has been shown to contribute to the quality of life in the physical, functional, and social domains (35). Increasing muscle and joint strength should be highly recommended for older adults since it effectively prevents falls and improves their quality of life (10). Walking as an aerobic exercise also positively affects quality of life (1). Various community exercise programs should be promoted, and they should consider the intensity and frequency of exercises for adequate physical activity.

In this study, multiple regression analysis showed that normal BMI was the most influential factor on the HRQoL of older women with injuries. In previous studies, the low BMI of older women was reported to be associated with low HRQoL (34, 35). Some studies also found that the more time a person spends sitting or lying down or the more severe their degree of obesity, the lower their quality of life (1, 16). In this study, especially in the lower BMI (underweight) group, the HRQoL was significantly lower than that in the higher BMI (overweight) group, which is consistent with the results of previous studies. Therefore, there should be increased awareness of the importance of maintaining a normal BMI in the older population. Previous studies on the association of being slightly overweight with HRQoL reported somewhat conflicting results (36, 37); thus, further research is needed for clarification.

Multiple regression analysis showed that stress and poor subjective health status were related to the low HRQoL of older women with injuries. These findings are consistent with the results of previous studies (16, 28). Stress during older adulthood is related to various diseases, economic difficulties, and reduced social activity. Stress is a mental health indicator that positively correlates with depression (32). Similar to this study, stress has been reported as a factor influencing the quality of life of older adults in previous studies (29, 32). We encourage continuing efforts to study the stressors of older adults with injuries and to provide them with healthcare services and health-related welfare support. Subjective health status is a significant index for predicting the prevalence and mortality of physical and mental diseases (38). Previous studies have supported the observation that stress is a key factor that influences the HRQoL of vulnerable older adults, such as older adults with low income and seniors living alone (10, 16). Economic activity is the most influential factor in improving older adults’ quality of life by enhancing their physical and mental satisfaction through social participation and satisfying their basic living needs (29). Supporting the economic activities of older adults can help them avoid social isolation and economic marginalization and live healthier lives. A national-level jobs program that provides stable income, maintains and expands individual skills, and guarantees continuous voluntary participation could support social activities for older women. For older adults with injuries, individualized approaches are needed to promote various social and physical activities based on periodic assessments of physical, emotional, and spiritual health conditions and consideration of the severity of their injuries. A large-scale nationwide survey on the factors influencing the HRQoL of older women with injuries may lead to the creation of a database that can be used for the future development of health-related programs.

This study has several limitations. Since this was a cross-sectional study that used secondary data analysis, we cannot explain the temporal context and related factors for older women with injuries. In this study, we did not consider other illnesses, the severity and duration of the injury, the treatment period, the site of the injury, or whether surgery was required to treat the injury. Further studies should closely observe the physical and mental health and the environmental factors that may affect older women.

## Conclusion

This study identified factors influencing the HRQoL of older women aged 65 years or over with injuries using KNHANES data. Based on the results of the study, the HRQoL of older women with injuries was significantly lower than that of older women without injuries. Unemployment, no physical activity, underweight, overweight, stress, osteoarthritis, and poor subjective health status were significantly associated with the low HRQoL of older women with injuries. This study has limitations as it was a cross-sectional study and can only provide limited contextual information on how the injury experience affects the HRQoL. Additionally, the data used for the analysis could not distinguish the severity of the injuries or diseases. Despite these limitations, this study is significant since it used a representative sample from a nationwide database, the KNHANES. The results of this study can support future research and assist in the development of health-related programs for older women with injuries.

## Data availability statement

The datasets used and/or analyzed during the current study are publicly available upon request via https://knhanes.kdca.go.kr/knhanes/sub03/sub03_02_05.do.

## Ethics statement

The studies involving human participants were reviewed and approved by Korea Disease Control and Prevention Agency. The patients/participants provided their written informed consent to participate in this study.

## Author contributions

YK and J-WA contributed to study conception, design, data analysis, and interpretation of the data. The first draft of the manuscript was written by YK. The manuscript has been critically revised by J-WA for important intellectual content. All authors contributed to the article and approved the submitted version.

## Funding

This research was supported by the Chung-Ang University Research Grants in 2021.

## Conflict of interest

The authors declare that the research was conducted in the absence of any commercial or financial relationships that could be construed as a potential conflict of interest.

## Publisher’s note

All claims expressed in this article are solely those of the authors and do not necessarily represent those of their affiliated organizations, or those of the publisher, the editors and the reviewers. Any product that may be evaluated in this article, or claim that may be made by its manufacturer, is not guaranteed or endorsed by the publisher.
